# Dopamine boosts intention and action awareness in Parkinson’s disease

**DOI:** 10.1007/s00221-020-05847-2

**Published:** 2020-06-27

**Authors:** Steven Di Costa, Ewgenia Barow, Ute Hidding, Tina Mainka, Monika Pötter-Nerger, Carsten Buhmann, Christian K. E. Moll, Patrick Haggard, Christos Ganos

**Affiliations:** 1 Institute of Cognitive Neuroscience, University College London (UCL), London, UK; 2 Department of Neurology, University Medical Center Hamburg-Eppendorf (UKE), Hamburg, Germany; 3 Department of Neurology, Charité University Medicine Berlin, Charitéplatz 1, 10117 Berlin, Germany; 4 Department of Neurophysiology and Pathophysiology, University Medical Center Hamburg-Eppendorf (UKE), Hamburg, Germany

**Keywords:** Parkinson’s disease, Intention awareness, Action awareness, Dopamine, Libet

## Abstract

Dopaminergic deficiency in Parkinson’s disease (PD) has been associated with underactivation of the supplementary motor area and a reduction of voluntary actions. In these patients, awareness of intention to act has been shown to be delayed. However, delayed awareness of intention to act has also been shown in patients with hyperdopaminergic states and an excess of unwilled movements, as in Tourette’s, and in patients with functional movement disorders. Hence, the role of dopamine in the awareness of intention and action remains unclear. 36 PD patients were tested ON and OFF dopaminergic medication and compared with 35 healthy age-matched controls. In addition, 17 PD patients with subthalamic deep brain stimulation (DBS) were tested ON medication and ON and OFF stimulation. Participants judged either the moment a self-generated action was performed, or the moment the urge to perform the action was felt, using the “Libet method”. Temporal judgments of intention and action awareness were comparable between unmedicated PD patients and controls. Dopaminergic medication boosted anticipatory awareness of both intentions and actions in PD patients, relative to an unmedicated condition. The difference between ON/OFF DBS was not statistically reliable. Functional improvement of motor ability in PD through dopaminergic supplementation leads to earlier awareness of both intention, and of voluntary action.

**Electronic supplementary material:**

The online version of this article (10.1007/s00221-020-05847-2) contains supplementary material, which is available to authorized users.

## Introduction

The initiation of voluntary actions relies on a well-organized network of neural structures that evaluate the salience of a selected motor program and allow its timely execution (Haggard 2008). In neurological disorders, different pathologies can disrupt this process and thereby lead to motor deficits related to inappropriate execution timing. For example, lesions of the dorsolateral prefrontal cortex have been associated with the inability to inhibit (pre-potent) actions (Aron et al. 2004), whereas acute structural damage of the supplementary motor area (SMA) will typically lead to a disruption or even complete loss of the capacity to generate voluntary motor output (Brugger et al. 2015). Slowness of action initiation, a defining feature of Parkinson’s disease (PD), results from reduced dopaminergic availability in the nigrostriatal pathway, characteristic of the neurodegenerative process in PD (Berardelli et al. 2001).

Several studies have linked deficits in voluntary action to reduction in basal ganglia–cortical drive as a result of dopamine deficiency in PD (Magrinelli et al. 2016). For example, the build-up of the Readiness Potential was found to be reduced and delayed in PD patients, compared to healthy volunteer controls (Berardelli et al. 1989; Marsden et al. 1995). Interestingly, this physiological signal has also been widely associated with the subjective experience of intention and volition. For example, Libet et al. showed that the subjective experience of the intention to move followed the gradual build-up of the Readiness Potential (Libet 1983). Therefore, one might expect that the reduced physiological drive in action initiation circuits in PD might be accompanied by an altered subjective experience of volition. However, clinical experience does not clearly favor this view—patients typically express the wish to move, but lack the motor capacity to do so—and indeed, the relation between the timing of the Readiness Potential and the timing of conscious intention to act remain unclear (Matsuhashi and Hallett 2008).

One previous study reported that PD patients show significant delays in the awareness of their intention to act (Tabu et al. 2015). However, the study sample was relatively small (*n* = 13 for each group: patients and controls) and detailed effects of disease severity and dopaminergic state (medication ON/OFF) could not be examined. Indeed, disease severity was heterogeneous in the patient group (Hoehn and Yahr stages 1–4) and all patients were examined in dopaminergic OFF condition only. Further, intention awareness is also delayed in hyperkinetic movement disorders––including Tourette syndrome (Baek et al. 2017; Edwards et al. 2011; Moretto et al. 2011), a disorder putatively characterized by a hyperdopaminergic state (Maia and Conceicao 2018)––challenging the idea that late awareness of intention is a direct and specific consequence of dopamine deficiency. The exact role of dopamine in the awareness of intentions thus remains unclear.

In the present study, we sought to explore temporal judgments of intention and action awareness in a large sample of PD patients and age-matched healthy controls. We specifically investigated the influence of PD pathology and dopaminergic intervention. An additional group of PD patients with subthalamic deep brain stimulation (DBS) was also examined to assess the effects of stimulation. There were two key hypotheses. First, if delayed intention awareness is a general feature of pathology, then patients OFF intervention should show delayed awareness relative to healthy controls (Tabu et al. 2015). Second, improvement of motor performance via restoration of dopaminergic availability through pharmacology or functional intervention (DBS) should influence awareness of intention and of action.

## Methods

### Participants

This experiment conformed to the Declaration of Helsinki and was approved by the Hamburg Ethics Committee. All participants agreed to participate in the study and signed a consent form. They all reported normal or corrected to normal vision and hearing. In total, 40 medicated PD patients (medication group: 27 males) with a mean age of 58 ± 8 (standard deviation; SD) and further 20 PD patients with DBS (DBS group: 13 males) and a mean age of 65 ± 7 (SD) attending the Department of Neurology of the University Medical Centre Hamburg-Eppendorf agreed to participate and were included in the study. Thirty-five healthy, age-matched [10 males, 59 ± 10 years (mean age ± SD)] volunteers were also included in the study and served as a control group.

Inclusion criteria for patients were a diagnosis of PD according to the Movement Disorders Society clinical diagnostic criteria and stable treatment and clinical condition for at least 4 weeks prior to the study (Postuma et al. 2015). For all patients, the following demographic and clinical data were collected: Hoehn and Yahr disease severity score (Hoehn and Yahr 1967), motor impairment in both OFF and ON medication conditions according to the Unified Parkinson’s Disease Rating Scale Part 3 motor examination (UPDRS-III), and levodopa (l-dopa) and dopamine-agonist (DA) equivalent daily dose (LEDD; Tomlinson 2010) and impulsive behavior according to the Questionnaire for Impulsive–compulsive Behavior Disorders in Parkinson's disease (QUIP-RS; Weintraub et al. 2012). For all participants, the Montreal Cognitive Assessment (MOCA; Nasreddine et al. 2005) was employed to assess overall cognitive function and the Multiple Features Target Cancellation (MFCT; Marra et al. 2013) for selective visual attention. All participants completed the Beck Depression Inventory (BDI-II; Beck et al. 1996).

Exclusion criteria for all groups were any major concurrent neurological or psychiatric disorders (no exclusions), a MOCA score < 26 (one exclusion was made in the DBS group) or a BDI-II score of 16 or more (three exclusions were made in the medication group and two exclusion were made in the DBS group). One participant in the medication group was excluded due to a self-declared inability to understand the task. We, therefore, analyzed data from 36 medication patients, 17 DBS patients and 35 controls.

### Procedure

Participants were seated at a standard computer keyboard and screen. They fixated a clock with a single rotating hand. The clock diameter was 20 mm and the hand rotated every 2560 ms. At a time of their choosing, but after waiting for at least one full clock rotation, participants pressed the space bar. The clock continued to rotate for a random interval (between 1500 and 2500 ms) and then stopped. According to condition, participants were then prompted to judge the position of the clock hand either at the moment they pressed the space bar (M judgment), or at the moment they first experienced the urge to press the space bar (W judgment). Participants reported their judgments verbally and the experimenter entered the number using the keyboard.

At the start of the experiment, participants completed a short training block of each type of trial. This training block was repeated until participants were confident with the task. Participants then completed eight blocks of ten trials each. W and M judgments were assessed in half the blocks each, in pseudorandom order.

Control participants completed the experiment and underwent the clinical assessment only once. Patients completed the experiment twice, once while OFF intervention (medication or DBS) and once while ON. Medication OFF was defined as a withdrawal of all dopaminergic medication for at least 12 h. For a medication ON condition, patients were given an individually adapted dose of water-soluble l-dopa (Madopar LT) based on the previously determined dose needed to achieve “best medication ON” and limited by a maximum of 300 mg to avoid overdosing and dyskinesias. Medication group patients arrived at the experiment having not taken their prescribed medication for a period of at least 12 h. The experiment was then run for the first time, after which the patients underwent the clinical assessments. Patients were then administered medication and waited half an hour before undergoing a second clinical assessment and then completing the experiment again.

Intervention state was counterbalanced in the DBS group. The required waiting time for the DBS to change state (active/inactive) was set at half an hour. Due to the severity of motor deficits of participants with DBS, ON/OFF manipulation involved only DBS, leaving ongoing medication status unaffected (also see Online Resource 1).

All stimuli were presented using LabView 2012 (National Instruments, Austin, TX). To determine if there were any differences in the time of movement onset between patients and controls and within patients (OFF and ON medication), electromyogram (EMG) recordings were taken from the first dorsal interosseus muscle. EMG data were analyzed using AcqKnowledge (Biopac Systems Inc. CA, USA).

### Analysis

The dependent variable was calculated by subtracting the W or M judgment from the actual time of the space bar press, as captured by the program, and analyzed by factorial ANOVAs.

Our analyses were chosen to test separately the effects of pathology and intervention. First, to test for an effect of pathology, data from healthy participants and patients OFF medication were submitted to a 2 × 2 between-subject ANOVA with factors task (W or M judgment) and group (patient or control). We then tested for effects of intervention separately for medication and for DBS, with two within-subject ANOVAs with factors state (OFF/ON) and task (W/M).

EMG data from patients on medication and healthy controls were time-locked to the button press and averaged across all trials. The resulting curves were visually inspected for the time of movement onset by two independent researchers blind to the experimental aims and conditions. These results were then averaged across researchers to receive final estimates of movement onset for each participant.

## Results

Clinical characteristics of our studied sample are presented in Table 1. Change from OFF/ON due to dopaminergic supplementation or DBS was associated with a significant improvement of motor function as captured by the UPDRS-III as confirmed by individual repeated-measures *t* tests (medication group: *t*_(35)_ = 14.55, *p* < 0.00001; DBS group: *t*_(16)_ = 7.3, *p* < 0.00001).

**Table 1:** Clinical characteristics

Variable	Medication group (*N* = 36)	DBS group (*N* = 17)	Control group (*N* = 35)
Age,mean (SD), years	57.50 (8.33)	65.29 (7.28)	58.71 (10.41)
Sex, No. (%)			
Female	9 (25)	4 (23.53)	25 (71.43)
Male	27 (75)	13 (76.47)	10 (28.57)
MOCA, mean (SD)	28.03 (1.30)	28.24 (1.60)	28.29 (1.36)
BDI-II, mean (SD)	6.44 (3.62)	6.41 (4.42)	3.57 (3.79)
Disease duration, mean (SD), years	5.19 (3.23)	12.18 (5.47)	NA
Hoehn and Yahr Stage, mean (SD)	1.94 (0.53)	2.35 (0.46)	
UPDRS part III MED/DBS OFF, mean (SD)	26.25 (9.90)	33.03 (14.97)	
UPDRS part III MED/DBS ON, mean (SD)	17.17 (7.06)	19.18 (11.15)	
Dopamine dose, mean (SD), mg^a^	194.44 (50.40)	NA	
ICD, mean (SD)	6.67 (5.71)	4.31 (4.94)	
QUIP, mean (SD)	10.47 (8.74)	9.13 (8.75)	

*BDI* Beck Depression Inventory, *DBS* deep brain stimulation, *ICD* impulse control disorder, *MED* medication, *MOCA* Montreal Cognitive Assessment, *NA* not applicable, *QUIP* Questionnaire for Impulsive-Compulsive Disorders in Parkinson’s Disease, *SD* standard deviation, *UPDRS* Unified Parkinson’s Disease Rating Scale

^a^applied dopamine dose to achieve medication ON

### EMG analysis

There was a significant positive correlation of movement onset estimates across researchers (*r* = 0.75, *p* < 0.01). Analysis of these estimates showed that the moment of action onset prior to button presses did not differ significantly in PD patients between ON and OFF medication states (*t*_(62)_ = 0.015, *p* = 0.98, BF_01_ = 3.91). There was also no significant difference between onset times between patients OFF medication and healthy controls (*t*_(64)_ = 1.14, *p* = 0.26, BF_01_ = 2.28, see Online Resource 2). We, therefore, proceeded to analyze awareness of action and intentions on the assumption that the neural precedents of the physical movement were comparable across all participants.

**Fig. 1:**
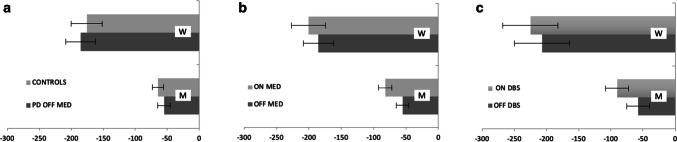
Perceived time of intentions (W) and actions (M) relative to actual movement onset comparing: **a** PD patients OFF medication and healthy controls. **b** PD patients OFF and ON medication. The OFF data is the same as in (**a**). **c** PD patients OFF and ON DBS. Error bars represent standard errors

First, we examined whether W and M judgments in the PD medication group OFF medication differed from healthy controls. ANOVA yielded a significant main effect of task, with W judgments earlier than M judgments (*F*_(1,138)_ = 44.43, *p* < 0.001, $\eta_{p}^{2}$ = 0.24, BF_01_ =  < 0.001). There was no significant effect of group (*F*_(1,138)_ < 0.001, *p* = *0.995*, $\eta_{p}^{2}$ < 0.001, BF_01_ = 5.81) and no interaction (*F*_(1,138)_ = 0.26, *p* = 0.61, $\eta_{p}^{2}$ = 0.001, BF_01_ = 3.46) (Fig. 1a). There was, therefore, no evidence that PD patients performed any differently to healthy controls. There was no correlation of either M and W judgments with disease severity in OFF (UPDRS-III). (M: *r* = − 0.11, *p* = 0.51; W: *r* = 0.05, *p* = 0.79).

Second, for the PD medication group, we examined the effects of dopamine (OFF/ON state) on W and M judgments using a within-subject ANOVA. A significant main effect of task was found, with W judgments earlier than M judgments (*F*_(1,35)_ = 31.2, *p* < 0.001, $\eta_{p}^{2}$ = 0.41, BF_01_ < 0.001). There was also a significant effect of medication state (*F*_(1,35)_ = 5.24, *p* = 0.028, $\eta_{p}^{2}$ = 0.01, BF_01_ = 2.04) but no interaction (*F*_(1,35)_ = 0.61, *p* = 0.44, $\eta_{p}^{2}$ < 0.001, BF_01_ = 4.1), suggesting that patients perceived both W and M events as occurring earlier while on dopaminergic supplementation (Fig. 1b). There was no correlation between the difference in M and W judgments (M–W) in the ON state and applied dopamine dose to achieve medication ON (*r* = − 0.03, *p* = 0.86).

Finally, we controlled whether DBS (DBS ON/DBS OFF) plays a specific role in W- and M-judgments. Here, within-subject ANOVA showed a strong effect of task, with W judgments earlier than M judgments (*F*_(1,16)_ = 18.13, *p* < 0.001, $\eta_{p}^{2}$ = 0.4, BF_01_ < 0.001), but there was no significant effect of state (*F*_(1,16)_ = 1.66, *p* = 0.22*,* $\eta_{p}^{2}$ = 0.01, BF_01_ = 2.56) and no interaction (*F*_(1,16)_ = 0.16, *p* = 0.69, $\eta_{p}^{2}$ = 0.01, BF_01_ = 2.98) (Fig. 1c). There was, therefore, no evidence that PD patients perceive the timing of their intentions and actions differently while ON than OFF DBS. Note, however, that the pattern of results is numerically similar to that of the medication group.

## Discussion

The results obtained in these experiments suggest that temporal judgments of intention and action awareness are comparable between PD patients and healthy controls. Furthermore, dopaminergic supplementation leads to a more anticipatory awareness of both intentions and actions in PD patients.

Previous research suggested that diminished dopaminergic drive to frontal areas reduces the capacity to make self-generated movements (Aron et al. 2004; Marsden et al. 1995). Similar results were recently reported in rodents (da Silva et al. 2018). Further, dopaminergic interventions have been shown to increase readiness potential (RP) amplitude (Berardelli et al. 2001). In line with this, we found a main effect of ON versus OFF intervention, with no interaction with intervention type (DBS or medication), or judgment type (M, W).

The absence of any significant difference in temporal judgments between PD patients and healthy controls aligns with clinical experience. Despite motor impairments, the intention to move is typically unaltered in patients, even if the translation of intention into action may be impaired (Nutt et al. 2011). Our data support this observation with an implicit measurement, which may avoid some of the cognitive biases that can influence explicit self-reports (Bandura 1989; Metcalfe and Greene 2007). Additionally, we found that PD patients were able to monitor the temporal aspects of movement preparation and execution with comparable precision to healthy controls.

These results are in contrast to Tabu et al. (2015), who found that awareness of intention was delayed in PD, while awareness of action itself was not. This discrepancy may be due to factors that differed between the two studies. As an example, the age of participants and the severity of PD may have influenced performance (participants in Tabu’s study were roughly 10 years older and more severely affected compared to our patient sample). A difference in temporal judgments may become more readily detected as disease severity increases, or simply with age. However, our data do not support this hypothesis, as no correlation between either W or M judgments and disease severity was observed in our sample of 36 patients with PD.

We found a clear effect of dopaminergic medication. Patients made significantly earlier W and M judgments while ON medication than while OFF. The observed shift in perception may reflect a hyper-dopaminergic state in patients tested ON medication. Since dopamine has strong effects on both attention and time perception (Meck 2005), a general change in perceived event timing might be expected when available dopamine is increased.

We did not find evidence for an effect of DBS on perceived time of events. This may be due to the continued presence of dopaminergic medication in the DBS group, which could be interpreted as a ceiling effect driven by medication since DBS patients also received dopaminergic medication. In addition, the variability of timing estimates across individual patients was higher in the DBS group than in either of the other two groups. Thus, we cannot make strong claims about the effect of DBS on action awareness in PD. Numerically, the effects of DBS and dopaminergic medication were comparable.

Our data could be interpreted in two ways. First, the earlier timing of awareness after intervention could reflect a change in perceptual detection of movement-related processes. Alleviating the motor symptoms of PD via dopaminergic supplementation may have resulted in a neural environment where the signals driving voluntary action emerge more readily from the general motor noise. These signals would thus become more detectable: participants might strategically lower a perceptual threshold for detecting these signals, without risking increased false positives. Alternatively, dopaminergic medication might change the way the participants use the clock to report the perceptual detection of these signals. According to the “prior entry” phenomenon (Spence et al. 2001), an attended event appears to happen earlier than an unattended event. Thus, if medication boosted attention to internal movement-related signals, W and M times would shift earlier, as we have found. Increasing attention to the clock would have the opposite effect.

Moore et al. (2010) reached somewhat similar conclusions from a study testing PD patients in a different experimental paradigm. Using an “intentional binding” task (Haggard et al. 2002; for a review, see Moore and Obhi 2012) to measure the sense of agency for voluntary actions and their outcomes, they found an effect of dopamine on the perceived times of both actions (a button press) and their subsequent effects (a tone). Participants reported that the perceived time of the tone was shifted towards the time of the button press, and that the perceived time of the button press was shifted towards the time of the tone it produced. This binding effect was comparable to healthy controls for PD patients OFF medication, but was significantly stronger for PD patients ON medication. These results are consistent with the interpretations of the present study, i.e. a dopamine boost might strengthen signal detection, and/or cause a shift of attention to internal processes, rather than external events. However comparing the two studies requires caution, as the experimental paradigms are slightly different, as are the theoretical constructs. Here we have studied the experiences of volition and action, rather than the experience of control over external events.

Our study faces some limitations. First, the DBS group was small (*n* = 17) compared to the medicated group (*n* = 36), and indeed for DBS no changes were made in concomitant intake of dopaminergic medication. Ideally, patients would have been investigated in complete OFF (medication/DBS) with sole manipulation of DBS. However, advanced PD precluded this possibility as indeed the severity of motor deficits precluded complete withdrawal from all interventions for this experimental condition. Therefore, we cannot exclude a ceiling effect in the DBS group due to concomitant dopaminergic medication. Second, medicated patients always completed the experiment while OFF medication first, and then again while ON medication. This leads to a potential confound between the medication factor and the order of testing. As dopaminergic medication takes time to achieve full efficacy or to diminish after cessation, this confound could only have been eliminated by running two separate experimental sessions in counterbalanced order with a rest stop of at least 24 h. To maintain a large sample size and reduce drop-out rates, we opted for a single-day testing. Indeed, order-related factors, such as fatigue, or loss of attention cannot, therefore, be ruled out as alternative explanations for the effects observed. However, the fact that inattentiveness has been previously associated with late rather than early W judgements strongly argues against this possibility (Cleeremans and Caspar 2015; Ganos et al. 2015).

We conclude that functional improvement of motor function through dopaminergic supplementation in PD is associated with a boost of both intention awareness and action awareness.

## Electronic supplementary material

Below is the link to the electronic supplementary material.

Supplementary file1 (DOCX 55 kb)

Supplementary file2 EMG readings Mean EMG readings from the first dorsal interosseous in each group, averaged across trials and participants, time-locked to a button press at 0ms. Vertical lines represent mean researcher estimates of action onset (averaged across participants within each group) (TIF 2473 kb)

## References

[CR1] Aron AR, Robbins TW, Poldrack RA (2004). Inhibition and the right inferior frontal cortex. Trends Cogn Sci.

[CR2] Baek K, Donamayor N, Morris LS, Strelchuk D, Mitchell S, Mikheenko Y, Yeoh SY, Phillips W, Zandi M, Jenaway A, Walsh C, Voon V (2017). Impaired awareness of motor intention in functional neurological disorder: implications for voluntary and functional movement. Psychol Med.

[CR3] Bandura A (1989). Human agency in social cognitive theory. Am Psychol.

[CR4] Beck AT, Steer RA, Brown GK (1996). Manual for the beck depression inventory-II.

[CR5] Berardelli A, Day BL, Marsden CD, Rothwell JC, Dick JP, Gioux M, Buruma O, Thompson PD, Benecke R, Cantello R (1989). The Bereitschaftspotential is abnormal in Parkinson’s disease. Brain.

[CR6] Berardelli A, Rothwell JC, Thompson PD, Hallett M (2001). Pathophysiology of bradykinesia in Parkinson’s disease. Brain.

[CR7] Brugger F, Galovic M, Weder BJ, Kagi G (2015). Supplementary motor complex and disturbed motor control––a retrospective clinical and lesion analysis of patients after anterior cerebral artery stroke. Front Neurol.

[CR8] Cleeremans A, Caspar EA (2015). “Free will”: are we all equal? A dynamical perspective of the conscious intention to move. Neurosci Conscious.

[CR9] da Silva JA, Tecuapetla F, Paixao V, Costa RM (2018). Dopamine neuron activity before action initiation gates and invigorates future movements. Nature.

[CR10] Edwards MJ, Moretto G, Schwingenschuh P, Katschnig P, Bhatia KP, Haggard P (2011). Abnormal sense of intention preceding voluntary movement in patients with psychogenic tremor. Neuropsychologia.

[CR11] Ganos C, Asmuss L, Bongert J, Brandt V, Munchau A, Haggard P (2015). Volitional action as perceptual detection: predictors of conscious intention in adolescents with tic disorders. Cortex.

[CR12] Haggard P (2008). Human volition: towards a neuroscience of will. Nat Rev Neurosci.

[CR13] Haggard P, Clark S, Kalogeras J (2002). Voluntary action and conscious awareness. Nat Neurosci.

[CR15] Hoehn MM, Yahr MD (1967). Parkinsonism: onset, progression and mortality. Neurology.

[CR16] Libet ML (1983). Expected versus actual random inbreeding: a reinterpretation of the random/nonrandom inbreeding formula. Hum Biol.

[CR17] Magrinelli F, Picelli A, Tocco P, Federico A, Roncari L, Smania N, Zanette G, Tamburin S (2016). Pathophysiology of motor dysfunction in Parkinson’s disease as the rationale for drug treatment and rehabilitation. Parkinsons Dis.

[CR18] Maia TV, Conceicao VA (2018). Dopaminergic disturbances in Tourette syndrome: an integrative account. Biol Psychiatry.

[CR19] Marra C, Gainotti G, Scaricamazza E, Piccininni C, Ferraccioli M, Quaranta D (2013). The Multiple Features Target Cancellation (MFTC): an attentional visual conjunction search test. Normative values for the Italian population. Neurol Sci.

[CR20] Marsden CD, Jahanshahi M, Brooks DJ, Brown RG, Jenkins IH, Passingham RE (1995). Self-initiated versus externally triggered movements. I. An investigation using measurement of regional cerebral blood flow with PET and movement-related potentials in normal and Parkinson’s disease subjects. Brain.

[CR21] Matsuhashi M, Hallett M (2008). The timing of the conscious intention to move. Eur J Neurosci.

[CR22] Meck WH (2005). Neuropsychology of timing and time perception. Brain Cogn.

[CR23] Metcalfe J, Greene MJ (2007). Metacognition of agency. J Exp Psychol Gen.

[CR24] Moore JW, Schneider SA, Schwingenschuh P, Moretto G, Bhatia KP, Haggard P (2010). Dopaminergic medication boosts action-effect binding in Parkinson’s disease. Neuropsychologia.

[CR14] Moore JW, Obhi SS (2012). Intentional binding and the sense of agency: a review. Conscious Cogn.

[CR25] Moretto G, Schwingenschuh P, Katschnig P, Bhatia KP, Haggard P (2011). Delayed experience of volition in Gilles de la Tourette syndrome. J Neurol Neurosurg Psychiatry.

[CR26] Nasreddine ZS, Phillips NA, Bedirian V, Charbonneau S, Whitehead V, Collin I, Cummings JL, Chertkow H (2005). The Montreal Cognitive Assessment, MoCA: a brief screening tool for mild cognitive impairment. J Am Geriatr Soc.

[CR27] Nutt JG, Bloem BR, Giladi N, Hallett M, Horak FB, Nieuwboer A (2011). Freezing of gait: moving forward on a mysterious clinical phenomenon. Lancet Neurol.

[CR28] Postuma RB, Berg D, Stern M, Poewe W, Olanow CW, Oertel W, Obeso J, Marek K, Litvan I, Lang AE, Halliday G, Goetz CG, Gasser T, Dubois B, Chan P, Bloem BR, Adler CH, Deuschl G (2015). MDS clinical diagnostic criteria for Parkinson’s disease. Mov Disord.

[CR29] Spence C, Shore DI, Klein RM (2001). Multisensory prior entry. J Exp Psychol Gen.

[CR30] Tabu H, Aso T, Matsuhashi M, Ueki Y, Takahashi R, Fukuyama H, Shibasaki H, Mima T (2015). Parkinson’s disease patients showed delayed awareness of motor intention. Neurosci Res.

[CR31] Tomlinson CL, Stowe R, Patel S, Rick C, Gray R, Clarke CE (2010). Systematic review of levodopa dose equivalency reporting in Parkinson’s disease. Mov Disord.

[CR32] Weintraub D, Mamikonyan E, Papay K, Shea JA, Xie SX, Siderowf A (2012). Questionnaire for impulsive-compulsive disorders in Parkinson’s disease-rating scale. Mov Disord.

