# Supplementary material for: Dopamine boosts intention and action awareness in Parkinson’s disease
Source: Exp Brain Res. 2020 Jun 27;238(9):1989–95. doi: 10.1007/s00221-020-05847-2 (PMC7438368; doi:10.1007/s00221-020-05847-2)
Supplement: Supplementary file 1 — Supplementary file1 (DOCX 55 kb) [file 221_2020_5847_MOESM1_ESM.docx]

**Online Resource 1** Individual patient data

| **Age/ Gender** | **Disease duration in years** | **Hoehn & Yahr Stage** | **UPDRS part III MED/DBS OFF** | **UPDRS part III MED/DBS ON** | **Dopamine dosis in mg** | **DBS parameters** | **Medication prior to study (daily dosis)** | **MOCA** | **BDI-II** | **ICD** | **QUIP** |
| --- | --- | --- | --- | --- | --- | --- | --- | --- | --- | --- | --- |
| 65/F | 1 | 2 | 27 | 17 | 200 | NA | Levodopa/Benserazide (300/75 mg), Pramipexole (0.52 mg) | 26 | 14 | 7 | 11 |
| 51/M | 6 | 2 | 23 | 9 | 300 | NA | Levodopa/Benserazide (250/62.5 mg), Pramipexole (2.09 mg), Rasagiline (1 mg) | 28 | 8 | 3 | 7 |
| 70/F | 7 | 1 | 29 | 13 | 150 | NA | Pramipexole (1.05 mg), Rasagiline (1 mg) | 29 | 1 | 0 | 0 |
| 73/M | 9 | 3 | 48 | 33 | 300 | NA | Levodopa/Benserazide (100/25 mg), Levodopa/Carbidopa/Entacapone (550/137.5/1200 mg), Ropinirole (16 mg) | 29 | 6 | 4 | 7 |
| 46/M | 2 | 2 | 24 | 17 | 200 | NA | Levodopa/Carbidopa (250/62.5 mg), Rotigotine (8 mg), Amantadine (450 mg) | 29 | 5 | 10 | 11 |
| 56/F | 2 | 1 | 30 | 21 | 100 | NA | Levodopa/Benserazide (75/18.75 mg), Procyclidine hydrochloride (7.5 mg) | 29 | 9 | 7 | 19 |
| 69/F | 5 | 2 | 43 | 28 | 200 | NA | Pramipexole (1.75 mg), Rasagiline (1 mg), Amantadine (150 mg) | 28 | 10 | 11 | 19 |
| 54/M | 9 | 3 | 31 | 21 | 200 | NA | Levodopa/Benserazide (100/25 mg), Levodopa/Carbidopa/Entacapone (250/62.5/1000 mg), Ropinirole (12 mg), Amantadine (300 mg), Safinamide (100 mg) | 27 | 7 | 12 | 21 |
| 60/M | 7 | 2 | 31 | 19 | 200 | NA | Levodopa/Benserazide (200/50 mg), Pramipexole (2.1 mg), Rasagiline (1 mg) | 28 | 7 | 8 | 12 |
| 58/M | 5 | 3 | 28 | 19 | 250 | NA | Levodopa/Benserazide (100/25 mg), Levodopa/Carbidopa/Entacapone (450/112/1000 mg), Pramipexole (3.15), Rasagiline (1 mg), Rotigotine (8 mg) | 28 | 3 | 2 | 3 |
| 62/M | 12 | 3 | 32 | 24 | 200 | NA | Levodopa/Benserazide (400/100 mg), Ropinirole (20 mg), Amantadine (300 mg), Rasagilin (1 mg) | 27 | 3 | 0 | 0 |
| 64/M | 2 | 2 | 18 | 14 | 100 | NA | Rasagiline (1 mg), Ropinirole (6 mg) | 28 | 6 | 0 | 3 |
| 53/M | 8 | 2 | 32 | 23 | 200 | NA | Levodopa/Benserazide (300/75 mg), Pramipexole (3.15 mg), Amantadine (300 mg), Procyclidine hydrochloride (12.5 mg) | 26 | 6 | 15 | 19 |
| 63/M | 3 | 2 | 31 | 20 | 200 | NA | Ropinirole (16 mg) | 28 | 4 | 20 | 25 |
| 53/M | 3 | 2 | 22 | 12 | 200 | NA | Levodopa/Benserazide (200/50 mg), Ropinirole (24 mg) | 28 | 11 | 18 | 18 |
| 55/F | 2 | 2 | 15 | 10 | 200 | NA | Levodopa/Benserazide (100/25 mg), Rasagiline (1 mg), Rotigotine (8 mg) | 30 | 13 | 9 | 25 |
| 49/M | 3 | 2 | 19 | 13 | 150 | NA | Levodopa/Benserazide (150/37.5 mg), Rasagiline (1 mg), Piribedile (150 mg) | 27 | 5 | 0 | 0 |
| 32/M | 1 | 2 | 23 | 15 | 200 | NA | Rasagiline (1 mg), Pramipexole (1.57 mg) | 27 | 8 | 3 | 3 |
| 62/M | 4 | 2 | 35 | 23 | 200 | NA | Levodopa/Benserazide (300/75 mg), Pramipexole (1.05 mg), Rasagiline (1 mg), Rotigotine (4 mg) | 26 | 5 | 10 | 14 |
| 63/M | 8 | 2 | 34 | 23 | 300 | NA | Levodopa/Benserazide (600/100 mg), Ropinirole (64 mg) | 29 | 3 | 0 | 0 |
| 73/M | 10 | 2 | 20 | 14 | 200 | NA | Levodopa/Benserazide (300/75 mg), Rasagiline (1 mg), Ropinirole (12 mg) | 26 | 1 | 0 | 0 |
| 55/F | 2 | 1 | 18 | 13 | 150 | NA | Levodopa/Benserazide (250/62.5 mg), Pramipexole (1.05 mg), Amantadine (150 mg), Procyclidine hydrochloride (50 mg) | 29 | 12 | 2 | 2 |
| 53/M | 4 | 2 | 27 | 20 | 200 | NA | Levodopa/Benserazide (200/500 mg), Ropinirole (12 mg), Amantadine (450 mg) | 30 | 4 | 12 | 12 |
| 57/F | 5 | 2 | 18 | 13 | 200 | NA | Rasagiline (1 mg), Ropinirole (16 mg) | 26 | 10 | 1 | 2 |
| 50/F | 3 | 2 | 19 | 12 | 150 | NA | Ropinirole (12 mg) | 29 | 2 | 0 | 0 |
| 60/M | 4 | 2 | 36 | 22 | 200 | NA | Levodopa/Benserazide (100/25 mg), Ropinirole (12 mg), Amantadine (400 mg), Biperiden (4 mg) | 29 | 3 | 6 | 13 |
| 57/M | 7 | 2 | 24 | 12 | 200 | NA | Levodopa/Benserazide (100/25 mg), Levodopa/Carbidopa/Entacapone (500/125/800 mg), Pramipexole (1.05 mg), Rasagiline (1 mg), Amantadine (450 mg) | 27 | 7 | 5 | 5 |
| 66/M | 3 | 2 | 38 | 25 | 150 | NA | Levodopa/Benserazide (300/75 mg), Pramipexole (3.15 mg), Rasagiline (1 mg) | 27 | 3 | 3 | 3 |
| 54/M | 6 | 3 | 29 | 20 | 200 | NA | Levodopa/Benserazide (400/100 mg), Pramipexole (1.05 mg), Ropinirole (12 mg) | 28 | 8 | 8 | 16 |
| 45/M | 3 | 1 | 7 | 4 | 200 | NA | Levodopa/Benserazide (300/75 mg), Pramipexole (2.26 mg), Rasagiline (1 mg) | 28 | 12 | 16 | 21 |
| 57/M | 6 | 2 | 21 | 15 | 200 | NA | Levodopa/Benserazide (600/150 mg), Rasagilin (1 mg), Procyclidine hydrochloride (150 mg) | 30 | 1 | 4 | 4 |
| 47/M | 1 | 1 | 10 | 4 | 150 | NA | Levodopa/Benserazide (250/62.5 mg), Pramipexole (2 mg), Rasagiline (1 mg) | 30 | 6 | 11 | 24 |
| 64/M | 11 | 3 | 51 | 34 | 300 | NA | Levodopa/Benserazide (400/100 mg), Piribedile (200 mg), Selegiline (10 mg) | 26 | 4 | 3 | 7 |
| 59/F | 13 | 2 | 22 | 16 | 100 | NA | Levodopa/Benserazide (250/62.5 mg), Rotigotine (6 mg) | 29 | 13 | 11 | 25 |
| 60/M | 7 | 2 | 21 | 15 | 200 | NA | Levodopa/Carbidopa (250/62.5 mg), Tolcapone (300 mg), Piribedil (150 mg), Rasagilin (1 mg), Amantadine (150 mg) | 30 | 6 | 4 | 4 |
| 55/M | 3 | 1 | 9 | 5 | 150 | NA | Pramipexole (1.05 mg), Rasagiline (1 mg) | 28 | 6 | 15 | 22 |
| 67/M | 10 | 3 | 45 | 30 | NA | R: 180 Hz, 60 μs, 3.6 mA L: 180 Hz, 60 μs, 4.2 mA | Levodopa/Benserazide (200/50 mg), Levodopa/Carbidopa/Entacapone (200/50/800 mg), Pramipexole (1.57 mg) | 28 | 13 | 8 | 18 |
| 53/M | 9 | 2 | 49 | 24 | NA | R: 174 Hz, 60 μs, 5.5 mA L: 174 Hz, 60 μs, 4.5 mA | Levodopa/Carbidopa/Entacapone (500/125/1000 mg) | 29 | 1 | 7 | 20 |
| 63/M | 11 | 2 | 25 | 18 | NA | R: 130 Hz, 60μs, 2.7 V L: 130 Hz, 60 μs, 1.8 V | Levodopa/Carbidopa/Entacapone (250/62.5/600 mg), Rasagiline (1 mg), Pramipexole (2.1 mg) | 28 | 3 | 11 | 20 |
| 62/M | 7 | 3 | 39 | 24 | NA | R: 170 Hz, 60 μs, 3.5 mA L: 170 Hz, 60 μs, 2.0 mA | Levodopa/Carbidopa/Entacapone (300/75/600 mg) | 30 | 11 | 2 | 6 |
| 56/M | 15 | 2 | 27 | 20 | NA | R: 160 Hz, 60 μs, 3.3 V L: 160 Hz, 60 μs, 3.5 V | Levodopa/Carbidopa/Entacapone (475/118.75/1200 mg), Rasagiline (1 mg), Rotigotine (8 mg) | 29 | 6 | 4 | 7 |
| 76/F | 11 | 3 | 67 | 44 | NA | R: 170 Hz, 60 μs, 3.0 mA L: 170 Hz, 60 μs, 3.5 mA | Levodopa/Benserazide (125/31.25 mg), Opicapone (50 mg), Safinamide (50 mg), Amantadine (50 mg) | 28 | 8 | 2 | 2 |
| 55/M | 9 | 2 | 26 | 16 | NA | R: 130 Hz, 60 μs, 3.0 V L: 130 Hz, 60 μs, 3.0 V | Levodopa/Carbidopa/Entacapone (800/200/1400 mg), Rasagiline (1 mg) | 26 | 12 | 3 | 10 |
| 62/M | 18 | 3 | 46 | 40 | NA | R: 180 Hz, 60 μs, 2.5 mA L: 180 Hz, 60 μs, 3.8 mA | Levodopa/Benserazide (300/75 mg), Pramipexole (2.1 mg), Amantadine (300 mg) | 25 | 4 | 5 | 5 |
| 66/M | 9 | 2 | 18 | 13 | NA | R: 130 Hz, 60 μs, 2.2 mA L: 130 Hz, 60 μs, 2.5 mA | Levodopa/Benserazide (200/50 mg), Levodopa/Carbidopa/Entacapone (400/100/800 mg) | 30 | 1 | 0 | 0 |
| 59/M | 9 | 2 | 29 | 14 | NA | R: 130 Hz, 60 μs, 1.5 mA L: 130 Hz, 60 μs, 4.8 mA | none | 29 | 4 | 17 | 19 |
| 63/F | 9 | 3 | 18 | 13 | NA | R: 174 Hz, 60 μs, 3.5 mA L: 174 Hz, 60 μs, 3.2 mA | Levodopa/Benserazide (100/25 mg), Levodopa/Carbidopa/Entacapone (800/200/1400 mg), Pramipexole (0.52 mg), Amantadine (200 mg) | 26 | 12 | 0 | 2 |
| 66/M | 23 | 2 | 19 | 3 | NA | R: 130 Hz, 60 μs, 3.0 V L: 130 Hz, 60 μs, 3.0 V | Levodopa/Benserazide (200/50 mg), Levodopa/Carbidopa/Entacapone (200/50/800 mg), Safinamid (100 mg), Amantadine (200 mg) | 26 | 4 | 0 | 0 |
| 75/M | 12 | 2 | 10 | 1 | NA | R: 130 Hz, 60 μs, 1.7 V L: 130 Hz, 60 μs, 2.5 V | Levodopa/Benserazide (600/125 mg), Opicapone (50 mg), Safinamide (100 mg), Ropinirole (8 mg) | 30 | 9 | 0 | 4 |
| 73/M | 4 | 3 | 42.5 | 21 | NA | R: 130 Hz, 60 μs, 2.0 mA L: 130 Hz, 60 μs, 2.7 mA | Levodopa/Benserazide (200/50 mg), Levodopa/Carbidopa/Entacapone (325/81.25/1200 mg), Safinamide (50 mg) | 30 | 4 | 1 | 6 |
| 76/M | 17 | 3 | 18 | 13 | NA | R: 180 Hz, 60 μs, 3.5 mA L: 180 Hz, 60 μs, 3.5 mA | Levodopa/Carbidopa/Entacapone (375/93.75/800 mg), Amantadine (300 mg), Piribedil (100 mg) | 29 | 2 | 0 | 0 |
| 72/F | 24 | 2 | 38 | 14 | NA | R: 180 Hz, 60 μs, 2.9 V L: 180 Hz, 60 μs, 2.9 V | Levodopa/Benserazide (225/56.25 mg), Opicapone (50 mg), Rasagiline (1 mg), Amantadine (100 mg), Ropinirole (12 mg) | 28 | 2 | 9 | 27 |
| 66/F | 10 | 2 | 45 | 18 | NA | R: 154 Hz, 60 μs, 4.5 mA L: 154 Hz, 60 μs, 2.0 mA | Levodopa/Benserazide (100/25 mg), Levodopa/Carbidopa/Entacapone (525/131.25/1200 mg), Pirebidil (150 mg), Selegiline (10 mg) | 29 | 13 | NA | NA |

BDI, Beck Depression Inventory; DBS, deep brain stimulation; F, female; ICD, impulse control disorder; L, left side; M, male; MED, medication; MOCA, Montreal Cognitive Assessment; NA, not applicable; QUIP, Questionnaire for Impulsive-Compulsive Disorders in Parkinson’s Disease; R, right side; SD, standard deviation; UPDRS, Unified Parkinson’s Disease Rating Scale.
