# Supplementary figures and images for: Dopamine boosts intention and action awareness in Parkinson’s disease
Source: Exp Brain Res. 2020 Jun 27;238(9):1989–95. doi: 10.1007/s00221-020-05847-2 (PMC7438368; doi:10.1007/s00221-020-05847-2)

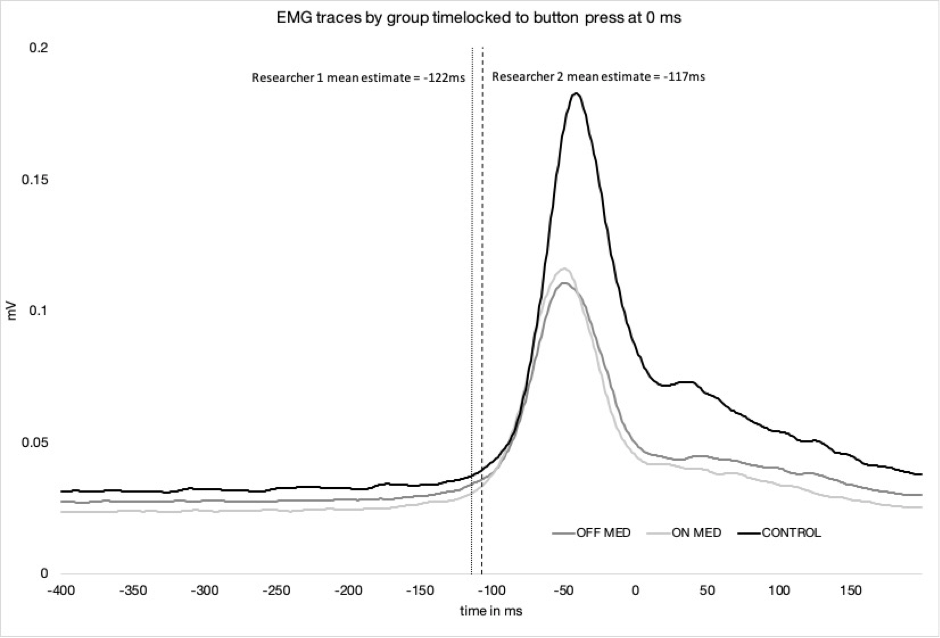

Supplement: Supplementary file 2 — Supplementary file2 EMG readings Mean EMG readings from the first dorsal interosseous in each group, averaged across trials and participants, time-locked to a button press at 0ms. Vertical lines represent mean researcher estimates of action onset (averaged across participants within each group) (TIF 2473 kb) [file 221_2020_5847_MOESM2_ESM.tif]
